# Image-guided, intensity-modulated radiotherapy for the treatment of diffuse-type tenosynovial giant cell tumor of the knee: Case report and review of the literature

**DOI:** 10.1097/MD.0000000000026659

**Published:** 2021-07-16

**Authors:** Xiaoyong Xiang, Wei Jiang, Chunyan Qiu, Nanjie Xiao, Jun Liang

**Affiliations:** Department of Radiation Oncology, National Cancer Center/National Clinical Research Center for Cancer/Cancer Hospital & Shenzhen Hospital, Chinese Academy of Medical Sciences and Peking Union Medical College, Shenzhen, China.

**Keywords:** case report, image-guided, intensity-modulated radiotherapy, radiotherapy, tenosynovial giant cell tumor

## Abstract

**Rationale:**

Tenosynovial giant cell tumor (TGCT) is a neoplastic, inflammatory disease with a benign but aggressive course that often presents as localized (TGCT-L) and diffuse (TGCT-D) forms based on the growth pattern and clinical behavior. For TGCT-L, simple excision of the diseased synovial tissue is the preferred treatment option, while for TGCT-D, adequate synovectomy is usually tricky but is essential. However, approximately 44% of TGCT-D cases will relapse after surgery alone. Thus, the optimal treatment strategy in patients with TGCT-D is evolving, and standalone surgical resection can no longer be regarded as the only treatment. The previous studies have shown that postoperative adjuvant radiotherapy can reduce recurrence in TGCT, especially in patients with incomplete synovectomy.

**Patient concerns:**

In the first case, a 54-year-old male presented with recurrent pain and swelling of the right knee with a protracted disease course (≥10 years). The other patient is a 64-year-old male who developed swelling, pain, abnormal bending, and limited movement of the left knee without obvious inducement.

**Diagnoses:**

Clinical and imaging examinations can provide a definitive diagnosis, and pathology is the gold standard. TGCT-D was confirmed by postoperative pathology. After the operation, the patients underwent an MRI re-examination and showed that the lesions of the knee were not completely resected.

**Interventions:**

Arthroscopic synovectomy was performed on the patients, and postoperative pathology was confirmed as TGCT-D. Because of incomplete synovectomy, the 2 cases received image-guided, intensity-modulated radiotherapy (IG-IMRT) after the operation.

**Outcomes:**

The follow-up time was 1 year, no evidence of disease progression was found in MRI. No obvious adverse effects associated with radiotherapy were detected during the follow-up period.

**Lessons:**

These cases and reviews illustrate the necessity of radiotherapy for TGCT-D and that IG-IMRT is a safe and effective method for treating TGCT-D of the knee.

## 1 Introduction

Tenosynovial giant cell tumor (TGCT) is an inflammatory and proliferative disease that includes previously known as a giant cell tumor of the tendon sheath and pigmented villonodular synovitis (PVNS).^[1,2]^ Clinically, TGCT often causes joint pain, swelling, stiffness, and limited range of motion. In 2013, the World Health Organization (WHO) reclassified TGCT into localized (local synovial involvement) and diffuse types (1 interarticular compartment or all intra-articular synovial involvement) according to the extent of the disease, with localized TGCT including nodular tenosynovitis and giant cell tumor of the tendon sheath, diffuse TGCT including PVNS and diffuse-type giant cell tumors.^[2]^ PVNS or diffuse-type synovial giant cell tumors (TGCT-D) are usually considered the same disease because of their identical histological and genetic features.^[3]^ The neoplastic and inflammatory characteristics of TGCT have been debated for many years.^[4]^ At present, TGCT-D is generally considered to be locally aggressive, which often affects tendon sheath, synovial bursa, and synovial tissue of large joints such as the knee, ankle, hip, temporomandibular, and elbow.^[5,26]^ They are all relatively rare soft tissue tumors, the annual incidence for TGCT has been estimated at 4 (TGCT-D) and 10 (TGCT-L, local-type synovial giant cell tumors) cases per million people, with a female predilection and the highest number of new possibilities in the age category 40 to 59 years.^[6]^

Currently, there is no agreement on the ideal treatment for patients with TGCT-D, the majority of patients receive arthroscopic and/or open surgical excision with synovectomy; however, the surgical approach is challenging to perform complete resection, and the recurrence rate can be as high as 44%, where recurrence after surgery was 2.6 times higher than those for TGCT-L.^[7,8]^ Because of the high rate of recurrence, researchers have been actively exploring alternative and adjuvant therapies. Radiotherapy has been recommended as an adjuvant to surgery or as the primary treatment for inoperable patients, however, some scholars have studied the efficacy and side effects of traditional radiotherapy techniques.^[9,10]^ From the literature, the common radiotherapy techniques included two-dimensional (2D-CRT) and three-dimensional conformal radiotherapy (3D-CRT), which might be related to the effect and side effects.^[10,20,24]^

The recent development of the image-guided technique (IGRT) in conjunction with intensity-modulated radiotherapy (IMRT) had significant clinical effects for better response and lower toxicity to the surrounding normal tissues.^[11]^ Here, we report 2 cases of TGCT-D in the knee after incomplete resection performed by image-guided intensity-modulated radiotherapy (IG-IMRT) and review the published studies.

## 2 Case presentation

### 2.1 Patient 1

A 54-year-old male presented with recurrent pain and swelling of the right knee with a protracted disease course (≥10 years). He had no other medical history and denied a family history of inherited diseases. He relieved the pain mainly through oral painkillers (specific unknown) and hot compress but did not received systemic therapies.

In November 2019, the patient's knee pain was aggravated after an accidental fall, and he went to the local hospital's orthopedic department. The clinician performed an MRI examination of the right knee joint and identified it as a synovial injury. The patient then underwent an arthroscopic synovectomy. During the operation, diffuse nodular hyperplasia of the whole joint's synovium seriously invaded the surrounding tissue. The surgeons tried their best to remove all synovial lesions, but they failed. Histological examination of the surgical specimens described characteristics compatible with TGCT-D, with areas of extensive necrosis and the presence of hemosiderin deposits.

The swelling and pain of the patient were slightly reduced 1 week after postoperation. Due to incomplete synovectomy and histological confirmation of TGCT-D diagnosis, the patient was referred to the radiotherapy department. Three weeks after the operation, the patient received further radiotherapy in the radiotherapy department. During the examination, the tenderness test of the medial joint space of the right knee was positive, the range of motion of the knee joint was limited, and the scar of the body surface operation had healed.

**Figure 1:**
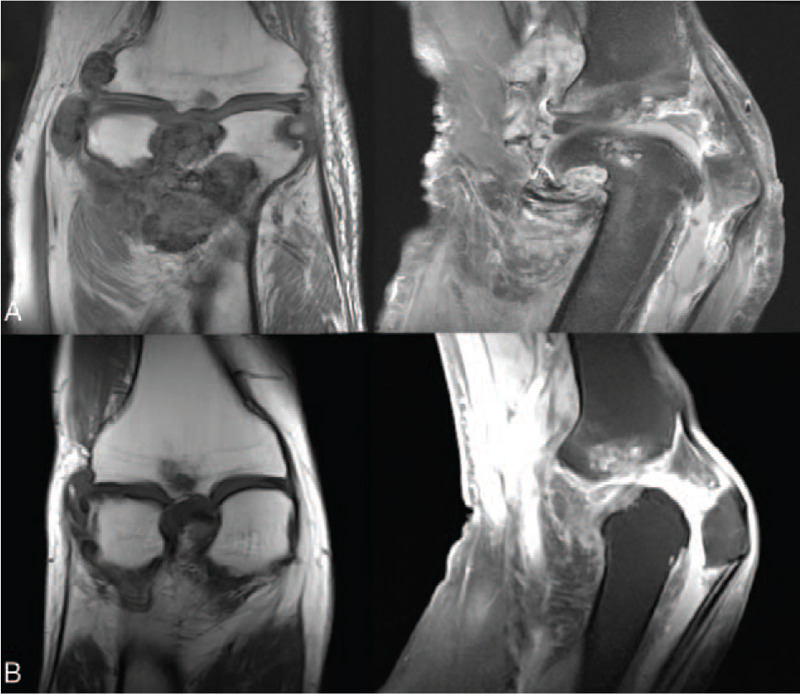
(A) Postoperative MR images demonstrate nodular and flocculent thickening of the right knee joint's synovium and the focus involved and invaded the surrounding tissue (patient 1). (B) Postoperative MR image showed that the left knee joint's synovium was uneven, widely thickened, and multiple nodular abnormal signals could be seen. (patient 2).

The patient underwent an MRI review after the operation. The right knee joint's synovium showed nodular and flocculent thickening, showing the mixed-signal intensity of T1WI and mixed high signal intensity of PDWI, and noticeable inhomogeneous enhancement in the enhanced scan. The focus locally involved and invaded the right patella, distal femur, proximal tibia and fibula, the articular surface of the right knee, adjacent bone destruction, absorption, bone marrow edema, etc. The focus involved and covered the right anterior and posterior cruciate ligament. MRI diagnosis showed that the lesions of the right knee joint were not completely resected. Figure 1A.

**Figure 2:**
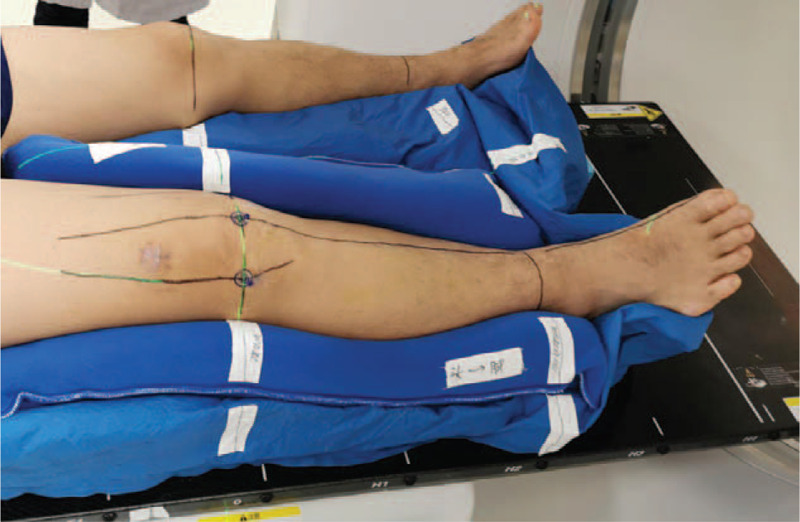
Patients were immobilized at the supine position with a vacuum bag with arms resting on the body's side and their legs separated. A combination of vacuum bags and thermoplastic pads is used to immobilize the patient's body position. At the time of simulation, a patient-specific immobilization device is used to ensure patient comfort and maximize reproducibility of the extremity's position.

After signing the informed consent for radiotherapy, a patient-specific immobilization device is used to ensure patient comfort and maximize the extremity position's reproducibility at the simulation time. Once the patient is in a satisfactory status and the immobilization is built, a CT scan is performed. The contralateral extremity position and other critical structures such as the genitalia were taken into account. The slice thickness of CT was 2.5 mm, and the scanning range included upper and lower 10 cm of knee joint lesions. Figure 2.

**Figure 3:**
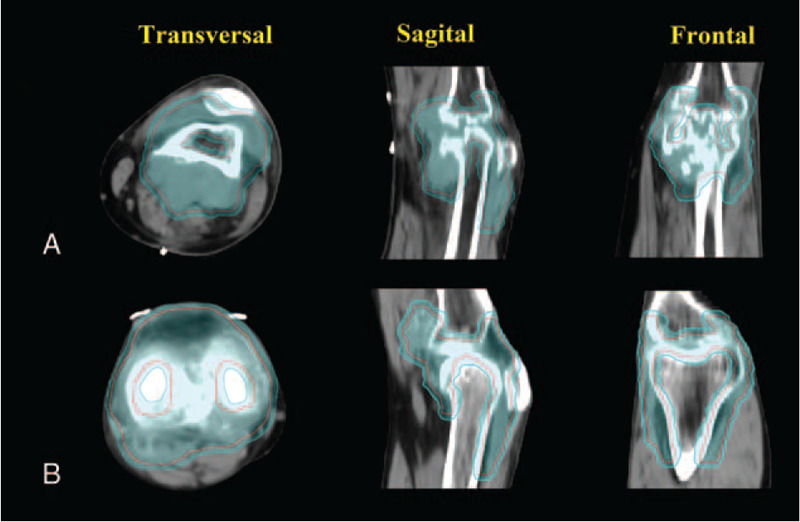
The diagnostic MRI merged with the treatment planning CT. The residual synovial lesions are outlined on the CT and MRI as the gross tumor volume (GTV). The clinical target volume (CTV) includes the whole knee joint cavity and GTV. The planning target volume (PTV) included the CTV with a 0.3 cm isotropic margin.

The diagnostic MRI merged with the treatment planning CT. The contrasted T1 and T2 series are the most useful. After resection, the residual synovial lesions are outlined on the CT and MRI as the gross tumor volume (GTV). The clinical target volume (CTV) includes the whole knee joint cavity and GTV. The MRI T2 peritumoral edema is usually included because of the risk of harboring microscopic extension of the tumor. The planning target volume (PTV) included the CTV with a 0.3 cm isotropic margin, outlined in cyan. Figure 3A. Normal tissue also has contoured, including normal bone, muscle, and soft tissue, which can be used as avoidance structures. PTV was administered 36 Gy in 18 daily fractions of 2Gy.

**Figure 4:**
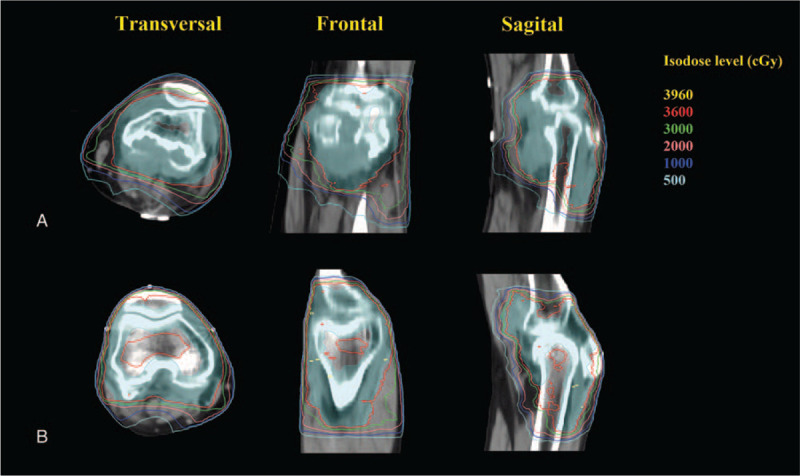
The radiotherapy plan used an 8-field IMRT technology, and the dose prescription is divided into 18 fractions by 36Gy. 95% of the PTV volume is required to receive the prescription dose. We limited the radiation dose to protect the normal tissues and avoided irradiating the limb's full circumference to prevent lower limb lymphedema.

Volumetric-based treatment planning is then performed using inverse planning for intensity-modulated radiation therapy (IMRT). It is taken to avoid irradiated the leg's entire circumference to decrease the risk of edema and joint stiffness. Figure 4A.

**Figure 5:**
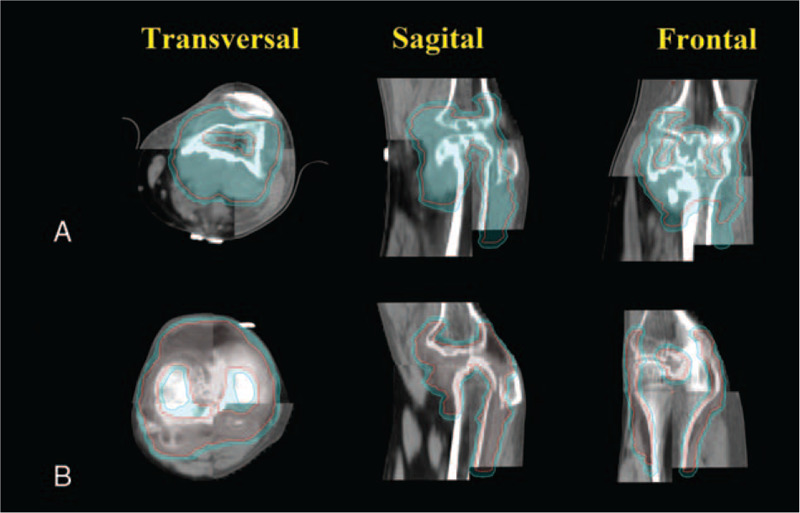
Image-guided radiotherapy (IGRT) could reduce errors caused by setup processes: before radiotherapy, the cone-beam CT (CBCT) images were used to register with the IMRT treatment plan images, and the position error was reduced to less than 1 mm.

At the time of radiotherapy, the patient is repositioned within the immobilization device in identical positions at CT simulation time. Linear accelerator performed a cone beam CT scan (CBCT) to verify soft tissues to confirm an accurate setup further. Daily IGRT is used to minimize the daily setup error. Figure 5A.

After 1 year postirradiation, no evidence of disease progression was found in MRI. No excess of obvious acute or chronic side effects such as joint activity limitation, skin necrosis, and malignant transformation was observed.

### 2.2 Patient 2

A 64-year-old male developed swelling, pain, abnormal bending, and limited movement of the left knee without obvious inducement in early October 2019.

MR examination was considered as patella malacia with soft tissue injury around the left knee joint and involving gastrocnemius muscle. The patient then underwent an arthroscopic synovectomy. During the operation, there was a large amount of bloody effusion in the left knee joint, diffuse nodular hyperplasia of the synovium, and severe invasion of the surrounding tissue. Postoperative pathology was confirmed as TGCT-D.

The swelling and pain of the patient were slightly reduced 1 week after postoperation. Due to incomplete synovectomy and histological confirmation of TGCT-D diagnosis, the patient received further radiotherapy.

Three weeks after the operation, the patient underwent an MRI re-examination and found that the left knee joint's synovium was uneven and widely thickened. Multiple nodular abnormal signals could be seen, showing the high signal intensity of T1WI, the low signal intensity of PDWI/FS, and the low signal intensity of DWI, consistent with TGCT-D. Figure 1B.

After signing the informed consent for radiotherapy, the patient then received further radiotherapy. CT simulation fixation method similar to patient one was used. The postoperative diagnostic MRI merged with the treatment planning CT. The residual synovial lesions are outlined on the CT and MRI as GTV. The CTV includes the whole knee joint cavity and GTV. PTV included the CTV with 0.3 cm isotropic margin (Fig. 3B). PTV was administered 36 Gy in 18 daily fractions of 2Gy. Volumetric-based treatment planning is then performed using inverse planning for IMRT (Fig. 4B). Daily IGRT is used to minimize the daily setup error (Fig. 5B).

After radiotherapy, there was only mild skin pigmentation around the knee, no obvious acute or chronic side effects, and no evidence of disease progression during the last follow-up time (December 2020).

## 3 Review of literature and discussion

TGCT is a neoplastic, inflammatory disease with a benign but aggressive course that often presents as localized and diffuse forms based on the growth pattern and clinical behavior, and the diffuse form is more common in clinical practice.^[1,2]^ For TGCT-L, simple excision of the diseased synovial tissue is the preferred treatment option, while for TGCT-D, adequate synovectomy is usually difficult but is essential.^[8,31]^ Previous studies have shown that postoperative adjuvant radiotherapy can reduce recurrence in TGCT-D, especially in cases with incomplete synovectomy.^[16,19,20]^ We reviewed the data from all available literature referring to adjuvant radiotherapy cases after surgery for the TGCT-D in the knee and discussed the characteristics.

TGCT is a relatively rare disease, and there is no consensus on its etiology and pathogenesis.^[1,2,32]^ Most scholars believe that TGCT is a non-tumoral reactive etiology, with models based on chronic inflammation, intra-articular repeated trauma and bleeding, or a possible implication of iterative microtrauma and lipid metabolism disorders.^[33,34]^ However, because of the distinct differences between the adjacent hyperplastic synovial tissue and the lesional tissue and the centrifugal growth pattern, other scholars considered that TGCT is a neoplastic process.^[35]^ In recent years, some researchers have found that overexpression of colony-stimulating factor-1 (CSF1) in TGCT cells leads to the recruitment of macrophages carrying CSF1 receptor (CSF1R) and translocation involving locus 1p 13.^[36,37]^

Among patients with TGCT, the most common site is the knee, less commonly the hips, ankles, shoulders, elbows, or temporomandibular joint.^[8,9,26]^ The main clinical manifestations include knee pain, swelling, stiffness, movement disorder, and deformation.^[7]^ Most patients with TGCT are characterized by hidden onset and a longer disease course, usually present with a history of trauma but no systemic symptoms. Clinically patients with TGCT are categorized into localized and diffuse subsets based on the extent of synovial involvement. X-ray or CT scan can only display nodular or lobulated mass in the joint, and they are difficult to determine the nature of the lesions. MRI has significantly higher soft-tissue resolution than X-ray / CT and was accurate and complete in displaying the location, extent, and depth of invasion of the lesions. On MRI, TGCT presents as a mass with lobulated contours margins and shown low signal intensity on both the T1- and T2-weighted images.^[38,39]^ Therefore, patients with TGCT should be implemented repeatedly by MRI at disease onset and during follow-up to observe dynamic changes in the lesions. However, the gold criteria for the diagnosis of TGCT need to be histopathologically diagnosed. Pathological specimens can be characterized that The hypertrophic synovium is typically villous, nodular, or villonodular and contains variable amounts of hemosiderin.^[40]^

The optimal treatment for TGCT is the total removal of the synovial lesions. Due to the location's heterogeneous nature and these lesions’ size, surgical modalities also markedly differed.^[8,41]^ For TGCT-L, complete surgical removal is advised in local lesions for the lower recurrence rate, while large excision is recommended for patients with bone infiltration. Previous studies have revealed that the complete removal of TGCT lesions can ensure no recurrence and radical cure.^[42,43]^ The optimal surgical treatment for TGCT-D is still a matter of debate, with combined anterior arthroscopic and open posterior excision being considered the preferred method.^[44]^ However, when the indications are not satisfied, knee arthroplasty should be performed as a limb-saving surgery to remove the diseased synovium.^[45]^ In summary, the treatment of TGCT-D should be individualized, and the significance of follow-up must be emphasized according to the specific location, the difficulty of local control, and the recurrence rate of surgical treatment. In recent years, with the development of minimally invasive technology and instruments, the surgical method of TGCT-D has gradually changed from open surgery to arthroscopic surgery. Arthroscopic surgery is an effective, minimally invasive surgical procedure that can narrow the scope of operation, reduce tissue trauma, and increase function allowing patients to improve their quality of life. However, arthroscopic surgery does not significantly reduce the recurrence rate of TGCT-D compared with open surgery. The largest study to date was a retrospective cohort study of 966 patients who underwent surgery alone. At a median follow-up of 54 months, the recurrent disease developed in 425 (44%) of all 966 surgically treated cases, and the 3 -, 5-, and 10-year recurrence-free survival rates were 62%, 55%, and 40%, respectively.^[8]^ It has even been found that the recurrence rate can be as high as 72% in TGCT-D of the knee after surgery alone.^[16]^ Thus, the optimal treatment strategy in patients with TGCT-D is evolving, and standalone surgical resection can no longer be regarded as the only treatment for patients with TGCT-D. With the subsequent discovery of neoplastic pathogenesis of TGCT, radiotherapy or macrophage-targeted agents, such as TNF-α and CSF1 R inhibitors, should also be considered in the clinic.^[46]^

Due to the anatomical limitation of the knee, radical resection of TGCT-D is very difficult, irrespective of surgical modalities. Postoperative adjuvant radiotherapy is often used, which can inhibit the proliferation of synoviocytes in the lining layer of the synovium, subsequently decrease the chance of a relapse. Moreover, low-dose radiotherapy can relieve bone and joint pain to a certain extent and positively affect the recovery of overall joint function after surgery. Currently, the radiotherapy techniques reported are radiosynoviorthesis and external beam radiotherapy (EBR), which can be used alone or as an auxiliary means of surgery, especially in the TGCT-D cases. Radiosynoviorthesis is the topical use of radioactive materials (most commonly 90yttrium) to restore the synovium as adjuvant or alternative therapy for surgery.^[5,13,14,25,29]^ However, the use of ROS has many disadvantages, including the potential risk of early-onset arthritis, avascular necrosis, difficult surgical wound healing, and other surgical complications, and requiring specialized radiotherapy delivery systems and surgery due to resource-intensive and invasive. EBR (such as 3-dimensional conformal radiation therapy and IMRT) is noninvasive and needs a widely used CT planning system and linear accelerator. At present, a large number of reports have confirmed that postoperative adjuvant external irradiation is an essential treatment for patients with TGCT-D, which can significantly improve local control.^[19–22,24,26,28]^ However, there is no consensus on the dose for postoperative radiotherapy. At present, most scholars believe that the total dose of 36Gy is lower than the long-term threshold of joint fibrosis, which is a safe and effective dose.^[47,48]^

In this study, we have performed a bibliographic search of PubMed in the past 10 years and presented the data of all TGCT-D cases which received adjuvant radiotherapy. For TGCT-D patients treated with surgery only, the recurrence rate was about 44% to 72%.^[8,16]^ Most of the TGCT-D patients who received ROS had a recurrence rate of between 23% and 44.1%. For patients who received postoperative traditional external beam radiation (EBRT) technology (including 2D-CRT, 3-dimensional conformal radiation therapy), different studies showed that the recurrence rate was between 0% and 17.4%. To summarize, the recurrence rate of patients who received adjuvant radiotherapy, especially EBRT, was significantly lower than that of patients with surgery alone (Table 1).

**Table 1:** Literature review of radiation used for the treatment of TGCT-D.

Author/year	Location and number (PORT group)	Study Period	Follow-up (months)	Surgery	Radiotherapy	Recurrence (%, n) or other prognostic information	Main toxicities of radiotherapy
Yang 2019^[12]^	TMJ: 1	2016	24	MiddleMiddle cranial fossa intracranial resection	IMRT: 40Gy/2Gy/20 F	0% (0/1)	There were no serious toxicities after PORT.
Dürr 2019^[13]^	Knee: 26	1996–2014	49	open synovectomy	RSO: 185 MBq (5 mCi) of ^90^Y-colloid was administered.	RSO: 23% (6 /26) Surgery-only: 27% (3/11)	Not reports.
Capellen 2018^[5]^	Knee: 29	1996–2014	71	open synovectomy	RSO: 185 MBq (5 mCi) of ^90^Y-colloid was administered. EBRT: Not detailed.	RSO: 32% (7/27) EBRT: 0% (0/2)	One case of femoral condylar necrosis occurred because of repeated ROS.
Gortzak 2018^[14]^	Knee: 34	1991–2014	87.6	Subtotal synovectomy	Adjuvant intra-articular injection of 90Yttrium	RSO: 44.1% (15 /34); Surgery-only: 50% (11/ 22)	Fourteen patients (41.2%) in the radiotherapy group developed degenerative changes at the x-ray.
Verspoor 2018^[15]^	TMJ: 2	2017	8-12	Irradical resection	EBRT: 45Gy/1.8Gy/25 F	No evidence of progressive disease.	There were no serious toxicities after EBRT.
Guo 2018^[16]^	Ankle: 26	2004–2015	54	Arthroscopic (10cases), Combined open synovectomy (16 cases)	6-MV X-ray beam: 20Gy/2Gy/10 F	Arthroscopic: 0%; Combined open synovectomy group: 31% (5/16).	No complications of nerve or vascular damage wound or joint infection, or deep venous thrombosis were observed in any patient.
Duan 2018^[17]^	Knee: 1	2014	36	Arthroscopic total synovectomy	3DCRT: 30Gy/2Gy/15F	0% (0/1)	No chronic toxic side effects such as joint activity limitation.
Serra 2017^[18]^	Shoulder: 1	2017	1	Partial arthroscopic synovectomy	15 and 6 MV photons: PTV∗ 40Gy/2.0Gy/20 F	0% (0/1)	Only an acute grade I radiodermatitis and a mild, painless swelling during radiotherapy.
Li 2015^[19]^	Knee: 26	2006–2011	54	Arthroscopic synovectomy	External radiotherapy: 20–30Gy/10F, once every other day. Two patients only completed 6Gy.	0% (0/26)	No complications, such as infection and vein thrombosis, were noted.
Mollon 2016^[20]^	Knee: 11	1998–2011	81	Combined synovectomies	EBRT: 35Gy/2.5Gy/14F; 40Gy/2Gy/20Gy+highest risk regions 8Gy/2Gy/4F	12% (2/11)	One patient developed femoral condyle AVN and one lymphedema
Safaee 2014^[21]^	TMJ∗: 2	2007–2013	28 and 6	One positive and one closed margin.	EBRT: 40Gy/2.0Gy/20 F and 45Gy/1.8Gy/25 F	0% (0/2)	There were no serious toxicities after EBRT.
Joshi 2015^[22]^	TMJ: 2	2010 and 2012	24 and 48	Incomplete synovectomy.	IMRT: 45Gy/1.8Gy/25 F	0% (0/2)	There were no serious toxicities after radiotherapy.
Bruns 2013^[23]^	Unclear location: 10	1980–2002	152.4	Unclear	RSO or RT	0% (0/10)^&^	Unclear
Park 2012^[24]^	Knee: 23	1998–2007	108	Unclear	4-MV or 6-MV EBRT: median dose 20 (12–34) Gy /10F	17.4% (4/23)	There were no serious toxicities (> grade 2) after radiotherapy.
Koca 2013^[25]^	Knee: 15	2006–2012	48±22	Arthroscopic synovectomy	RSO: 5 mCi ^90^Y citrate colloid was injected into the joints.	No disease progression was found in all patients.	Unclear
Griffin 2012^[26]^	Hand:6,Wris:4,Hip:4,Knee:20,Ankle:9, Foot:7	1972–2006	94	Unclear	EBRT (Cobalt-60, 4–6MV, 24–50Gy/13–25 F)	6% (3/50)	Two patients required subsequent total hip arthroplasty because of progressive osteoarthritis.
Carvalho 2012^[27]^	Knee: 8	1997–2008	103	Subtotal Synovectomy	15-MV linear accelerated photon beam: 20Gy (10–39.6Gy)/10F	12.5% (1/8)	Three patients had late minor complications.
Chen 2011^[28]^	Knee: 19	2001–2007	98	Unclear	EBRT: 30Gy/2.0Gy/15 F	10.5% (2/19); Residual tumor: 26.3% (5/19)	NO complications after radiotherapy.
Zook 2011^[29]^	Knee:8; Hip:1	Unclear	20	Unclear	Intra-articular chromic phosphate (^32^P)	30% (3/9)	During follow-up, there were no complications reported.
Judge 2011^[30]^	Ankle: 1	2003	96	Arthroplasty of the ankle joint	EBRT: 34Gy/15 F	0% (0/1)	Slight hyperpigmentation of the skin.

IMRT = intensity-modulated radiation therapy, PORT = postoperative radiotherapy, TMJ = temporomandibular joint, RSO = adjuvant radiosynoviorthesis, EBRT = external beam radiation, F = fraction, 3DCRT = 3-dimensional conformal radiation therapy, PTV∗, defined as the whole synovial space and residual lesion as evidenced by a preoperative MRI plus a margin of 1 cm, AVN = avascular necrosis, TMJ∗, the temporomandibular joint with intracranial extension, RT = radiation therapy; ^&^, ten patients who received ROS or RT after primary surgery did not show recurrence.

Despite concerns about serious radiotherapy complications, such as skin necrosis and malignant transformation, but previous studies have not found any malignant transformation cases or serious side effects following radiotherapy. None of the TGCT-D patients who were treated with EBRT experienced radiation-related early or late complications greater than grade 2, and none developed any radiation-induced malignancy. Only a few studies have reported mild radiation dermatitis, local pain, or occasional lymphedema in patients after EBRT.^[12,15–22,24,26,28]^ While in patients treated with ROS, there are less data relating to toxicities of radiotherapy. Only one study reported necrosis of the femoral condyle due to repeated ROS treatmen.^[5]^ Because ROS is resource-intensive and invasive, it requires specialized radiotherapy delivery systems and surgical procedures. However, EBRT is noninvasive and only needs the widely used CT planning system and linear accelerator. Thus, EBRT technology is more commonly used in the clinic for TGCT-D. The serious complications caused by low-dose radiation therapy are unlikely to occur. Furthermore, with the development and popularity of advanced radiotherapy techniques, such as three-dimensional conformal radiation therapy (3D-CRT), IMRT, IGRT, proton beam radiotherapy (PBT), stereotactic body radiotherapy (SBRT), and other precise radiation therapy technologies, the side effects of radiotherapy are further reduced.

Although examples of external irradiation for TGCT-D have been reported, the data available for the utility of IG-IMRT for TGCT-D of the knee is limited. IG-IMRT has the following advantages: highly conformal radiation doses can be selectively delivered to the clinical target volume while sparing the surrounding normal tissues; simultaneously, the daily setup error can be minimized by using daily image-guided. Here, we report 2 cases of TGCT-D in the knee after an incomplete synovectomy performed by IG-IMRT. After one year of postirradiation, no evidence of disease progression was found in MRI. No obvious acute or chronic side effects such as joint activity limitation, skin necrosis, and malignant transformation associated with radiotherapy were detected during the follow-up period.

## 4 Conclusions

In conclusion, in the era of multimodality therapy, standalone surgical resection can no longer be regarded as the only treatment for patients with TGCT-D. Postoperative adjuvant radiotherapy or combined approaches should be considered. IG-IMRT is a safe and effective method for the treatment of TGCT-D in the knee after incomplete synovectomy.

## Author contributions

**Data curation:** Wei Jiang.

**Formal analysis:** Wei Jiang.

**Investigation:** Wei Jiang, Chunyan Qiu, Nanjie Xiao, Jun Liang.

**Writing – original draft:** Xiaoyong Xiang, Jun Liang.
